# *Candida albicans* Targets a Lipid Raft/Dectin-1 Platform to Enter Human Monocytes and Induce Antigen Specific T Cell Responses

**DOI:** 10.1371/journal.pone.0142531

**Published:** 2015-11-12

**Authors:** Valeria de Turris, Raffaela Teloni, Paola Chiani, Carla Bromuro, Sabrina Mariotti, Manuela Pardini, Roberto Nisini, Antonella Torosantucci, Maria Cristina Gagliardi

**Affiliations:** 1 Department of Infectious, Parasitic and Immune mediated Diseases, Istituto Superiore di Sanità, 00161, Rome, Italy; 2 Center for Life Nanoscience, Istituto Italiano di Tecnologia, 00161, Rome, Italy; 3 Institute of Molecular Biology and Pathology, CNR, 00185, Rome, Italy; University of Birmingham, UNITED KINGDOM

## Abstract

Several pathogens have been described to enter host cells via cholesterol-enriched membrane lipid raft microdomains. We found that disruption of lipid rafts by the cholesterol-extracting agent methyl-β-cyclodextrin or by the cholesterol-binding antifungal drug Amphotericin B strongly impairs the uptake of the fungal pathogen *Candida albicans* by human monocytes, suggesting a role of raft microdomains in the phagocytosis of the fungus. Time lapse confocal imaging indicated that Dectin-1, the C-type lectin receptor that recognizes *Candida albicans* cell wall-associated β-glucan, is recruited to lipid rafts upon *Candida albicans* uptake by monocytes, supporting the notion that lipid rafts act as an entry platform. Interestingly disruption of lipid raft integrity and interference with fungus uptake do not alter cytokine production by monocytes in response to *Candida albicans* but drastically dampen fungus specific T cell response. In conclusion, these data suggest that monocyte lipid rafts play a crucial role in the innate and adaptive immune responses to *Candida albicans* in humans and highlight a new and unexpected immunomodulatory function of the antifungal drug Amphotericin B.

## Introduction

Lipid rafts are small, highly dynamic and tightly ordered plasma membrane microdomains, enriched in cholesterol, glycosphingolipids, glycosylphosphatidylinositol (GPI)-linked proteins and signaling-related molecules, that play a major role in regulation of protein sorting and organization within cell membranes [1–4]. In particular, lipid rafts can establish specialized membrane clusters where diverse cellular receptors are co-localized, concentrated and segregated with partners of their downstream signaling pathways and are crucially involved in coordination of cell signal transduction [4–6].

In recent years, raft microdomains have emerged as crucial surface platforms through which several bacterial, protozoan and viral pathogens can interact with host phagocytes to trigger or modulate the early anti-infectious innate immune response and the ensuing adaptive immune response [7–10]. Several receptors involved in microbe recognition by professional phagocytes stably or transiently reside in lipid rafts and initiate their signal cascades in these regions upon activation by pathogen binding [5, 11, 12]. Proper functioning of rafts is strictly required to guide the recruitment and multimerization of pathogen recognition receptors (PRRs) in the so called “phagocytic synapse”, a raft-organized protein bundle where receptor-mediated signalling is initiated and a multiplicity of anti-infectious host responses are triggered [13–16].

Rafts, or raft-associated proteins/lipids, also appear to participate in orchestration of the intracellular traffic of phagosome vesicles, driving the delivery of ingested microbial pathogens to degradative or non-degradative intracellular compartments and, consequently, their intracellular fate and availability for antigen processing [17–19]. Consistent with these findings, there is increasing evidence in *ex-vivo* models of microbial infections that disruption or perturbance of rafts microdomains impacts dramatically on pathogen-phagocyte interactions and may translate into inhibition of microbial adhesion and internalization, altered intracellular trafficking and killing of the pathogen as well as into modulation of the expression of various antimicrobial intermediates and cytokines [10, 20].

Finally, centrality of lipid rafts for host anti-infectious defence is also corroborated by the evidence that several microbial pathogens have evolved strategies to circumvent raft-mediated activation of phagocytes, such as the ability to subvert raft-associated signalling pathways or to co-opt raft microdomains as an entry portal to escape the intracellular, degradative lysosomal pathway [14].

For these reasons, lipid rafts have been recently the focus of an intense research work aimed to gain insights into molecular mechanisms of anti-infectious immune activation, also in view of the possible development of novel, raft-centered antimicrobial therapeutics or immune interventions.

There are very few information on whether fungi could exploit lipid rafts of human phagocytic cells to initiate or modulate the antifungal immune responses [21], although fungal pathogens remain a leading cause of highly lethal infections in immunocompromised individuals and in immunocompetent, hospitalized patients [22, 23].

To interact with the host immune system, *Candida albicans* (*C*. *albicans*) can target a disparate array of PRRs on the surface of human professional phagocytes [24, 25]. Toll-like receptors and C-type lectin receptors together with other fungal-relevant receptors, including FcγR, CR3, CD36, SCARF1 [26] and lactosylceramide (LacCer) [27–31] sense simultaneously several *C*. *albicans* pathogen associated molecular patterns (PAMPs) to orchestrate the so-called “PRRs crosstalk”. In neutrophils, LacCer-enriched lipid rafts have been involved in fungal β-glucan-driven chemotaxis and generation of superoxide [31, 32] as well as in phagocytosis of fungal β-glucan particles [33]. However, it is not known whether and to what extent raft activity is relevant in receptor-mediated initiation of the early responses of monocytes to fungal cells and in the ensuing specific adaptive immune response. In this study, we analyzed the role of lipid raft in *C*. *albicans* phagocytosis by human monocytes and the possible consequences of lipid raft disruption on the initiation of an anti-*C*. *albicans* specific immune response.

## Materials and Methods

### Ethics Statement

Specific approval of the local ethic committee was obtained for this study (Istituto Superiore di Sanità Prot. CE/13/386). A written informed consent was obtained from all participants.

### Reagents and monoclonal antibodies

RPMI 1640 and DMEM without PhenolRed (Euroclone ltd, UK) were used supplemented with 1mM L-glutamine, 1mM sodium pyruvate, 1% non-essential amino acids, 1% kanamicin and 1% heat inactivated fetal bovine serum (FBS) or 5% heat inactivated human serum (Hyclone, Logan, UT).

Deoxycholate Amphotericin B (DAmb), methyl-β-cyclodextrin (MBCD), laminarin, mannan, N-acetyl-D-glucosamine (GlcNAc) were purchased from Sigma-Aldrich S.r.l. (Milan, Italy).

AlexaFluor 647-conjugated B subunit of cholera toxin (Alexa647-CTB) was purchased from Life Technologies Italia (Monza, Italy).

Microparticulate, alkali-acid insoluble *C*. *albicans* β-glucan was obtained by repeated hot alkali and acid extraction of delipidized fungal cell walls. The extraction procedures consisted in a 24-h treatment with NaOH 1% (wt/v) T 100°C, followed by extensive washing (to neutrality) of the insoluble residue, and a second 24-h extraction with 0.5 M acetic acid at 80°C. After repeated washings, the alkali-acid insoluble β-glucan pellet was lyophilized and stored at +4°C. Chemical and 1H NMR analysis demonstrated that the preparation contained equal amounts of β-1,3- and β-1,6-linked glucan polymers, which accounted for 93% (wt/wt) of the preparation. FITC labeling of β-glucan particles (10 mg/ml) were performed as described for microbial cells (see next section)

The recombinant MP65 protein (from GeneScript Inc., NJ, USA) was cloned and expressed in poly-Histidine-tagged form in *Escherichia coli* according to its published sequence and purified by Nickel-chelate affinity chromatography.

PE-conjugated mouse monoclonal antibodies (mAbs) anti-human Dectin-1(clone 259931) and anti-CD14 (clone134620) were from R&D Systems (Minneapolis, MN), anti-CR3 (clone ICRF44), anti-MR (clone DCN46) and anti-DC-SIGN (clone 19.2) from BD-Bioscience (Milan, Italy).

### Microorganisms

*C*. *albicans* BP, serotype A, from the established type collection of the Istituto Superiore di Sanità, Rome, Italy, and *Staphylococcus aureus* strain ATCC25923 were used throughout this study. For monocyte stimulation the strains were grown overnight in Sabouraud’s dextrose broth at 28°C, or in broth medium (20 g/L tryptone, 5 g/L beef extract and 5 g/L NaCl) at 37°C, respectively. Microbial cells were collected by centrifugation, extensively washed and killed by heating at 65°C for 1h, followed by three freeze-thawing cycles for *S*. *aureus*. For fluorescein labeling, heat-inactivated (HK) microbial cells were resuspended in 0.01 mg/ml of FITC in 0.05 M carbonate-bicarbonate buffer (pH 9.5). After incubation for 15 min at room temperature in the dark, FITC-labeled cells were washed twice with PBS and resuspended to the appropriate concentration in the same buffer. The same procedures was used to label live Candida albicans cells.

### Isolation of human monocytes and T lymphocytes

This study has been approved by the Istituto Superiore di Sanità review board and written informed consent has been obtained by the healthy volunteers who participated to the study. PBMC from healthy donors were isolated by Ficoll density gradient. Monocytes were then positively sorted using anti-CD14-labeled magnetic beads (MACS, Miltenyi Biotech, Germany). Purity of cell population was controlled by flow cytometry analysis of CD14 expression (95–97% CD14^+^ cells). From the CD14^-^ fraction memory CD4^+^ T cells were negatively isolated by Memory CD4^+^ T cell Isolation Kit (MACS, Miltenyi Biotech, Germany), containing a cocktail of biotin-conjugated antibodies against CD8, CD14, CD16, CD19, CD36, CD45 RA, CD56, CD123, TCR γ/δ, Glycphorin A.

### Flow cytometry analysis of monocytes

Monocytes were stained at 4°C for 30 minutes with the following mAbs: anti-human CD14, Dectin-1, CR3, MR and DC-SIGN in PBS+1%FBS. After washing cells were collected and analyzed on a FACScan cytometer equipped with Cellquest Software (Becton Dickinson) acquiring 2x10^4^ events gated according to monocyte forward and size scatters.

### Phagocytosis analysis by flow cytometry

Monocytes were pre-treated or not with MBCD (5 mM) or DAmb (5 μg/ml) for 30 minutes at 37°C and then incubated for 1h at 37°C with FITC-conjugated heat killed or live *C*. *albicans*, *Staphylococcus aureus* at a ratio of 1:5 or with FITC-conjugated β-glucan (25 μg/ml) in RPMI+1% heat inactivated FBS.

In some experiments monocytes were pre-treated for 30 minutes at 37°C with laminarin, mannan, GlcNAc (100 μg/ml each) or with a purified mouse blocking mAb anti-human CD11b/Mac-1 (clone ICRF44, 50 μg/ml) before fungal stimulation.

The percentage of phagocytosing cells were examined on a FACScan cytometer equipped with Cellquest Software (Becton Dickinson) and determined by comparing the intensity of FITC before and after trypan blue (Sigma) quenching of membrane-bound microrganisms or β-glucan, leaving unchanged the signal coming from the engulfed ones. As a control, cells incubated with the microorganisms at 0°C were used to exclude background staining.

### Immunofluorescence and confocal imaging

Monocytes were plated on poly-Lysine-coated 8-wells ibiTreat μ-slide (ibidi), 2h before treatment. Drug treatments and phagocytosis of FITC-conjugated HK *C*. *albicans* was performed as described above. We performed the immunofluorescence (IF) directly on the chamber slide. Cells were fixed in 4% paraformaldehyde for 30 minutes at 4°C and washed twice with PBS. Fixed cells were then permeabilized with PBS containing 0.5% Triton X-100 for 5 minutes, blocked with 3% BSA and 0,05% Tween20 and then stained with anti-CD14 and Cy3-conjugated goat-anti mouse (Jackson ImmunoResearch). DAPI (Sigma-Aldrich) was used for nuclear counterstaining. Each well was mounted by filling with ibidi mounting medium (ibidi). Fluorescent and DIC (Differential Interference Contrast) images were acquired at the Olympus iX83 FluoView1200 confocal microscope with a 60x, NA1,35 objective. The following parameters were used: acquisition mode sequential, line average 2, zoom 6, z-step 0.5μm, 22 slices. Laser lines: 405 (1%), 473 (2%), 559 (5%). The orthogonal view and 3D rendering were performed using the Imaris image analysis software v.8.1.2.(Bitplane).

### Live cell imaging

Live monocytes were stained with Alexa647-CTB and anti-Dectin-1 for 30 minutes at 4°C, then with Cy3-conjugated goat-anti mouse (Jackson ImmunoResearch), washed, pre-treated or not with MBCD (5 mM) or DAmb (5 μg/ml) and then incubated for 30 minutes on ice with FITC-conjugated HK *C*. *albicans* at 1:3 ratio, to allow cell-cell interaction without phagocytosis. Cells were then resuspended in warm DMEM without PhenolRed supplemented with 1% FBS and 400.000 cells/well were plated on 8-wells ibiTreat μ-slide (ibidi) for time lapse imaging and acquisition started 10–20 min after plating. Cells were recorded for 2 hours. Fluorescent and DIC images were acquired at the Olympus iX83 FluoView1200 equipped with Olympus CellVivo incubation system to control temperature, humidity and CO_2_ conditions. The following settings were used: 60x, NA1,35 objective, acquisition mode sequential, no line average, zoom 3, single slice. Time series were acquired for 2 hours at 1 minute interval. Laser lines: 473 (0.4%), 559 (9%), 635 (15%).

### Cytokine determinations

Monocytes were seeded in 48-well cluster plates (5 × 10^5^ cells per well in 0.5 ml), pretreated or not for 30 minutes with DAmb (5 μg/ml) or MBCD (1 mM) and incubated at 37°C with or without *C*. *albicans* at a ratio of 1:5. Supernatants were collected after 18 hours incubation and frozen until use. IL-1β, TNF-α, IL-23, IL-6, IL-10 and IL-12 were measured by enzyme linked immunosorbent assay (ELISA) using commercially available kits (R&D Systems Europe, Ltd). Supernatants from the antigen presentation assays were collected after 6 days of culture and evaluated for IFN-γ and IL-17 production by ELISA. The tests were performed according to the manufacturer instructions. Detection limit of the assays was 15 pg/ml.

### Analysis of specific T cell response

Monocytes, isolated from donors highly responsive to *C*. *albicans*, pre-treated or not with MBCD (1 mM) or with DAmb (5 μg/ml) for 30 minutes and stimulated with *C*. *albicans* at a ratio of 1:1 for 2 hours were irradiated and plated onto 96 flat bottom well plate in RPMI+5% heat inactivated human serum. In some experiments monocytes were extensively washed after drug treatment and pulsing. Purified autologous CD4+ memory T cells were added at a ratio of 2:1 with monocytes. After 6 days of culture the proliferative response was measured by a 16-h pulse with ^3^H thymidine and cytokine production was analyzed by ELISA on culture supernatants.

### Statistical analysis

All the statistical analysis were performed using the fourth version of GraphPad Prism Software. The data were analyzed using the non-parametric Mann-Whitney *U* test. All tests for statistical significance were two-tailed and *p* values <0.05 were considered significant.

## Results and Discussion

### 1. Disruption of lipid rafts inhibits the uptake of *C*. *albicans* by human monocytes

**Fig 1:**
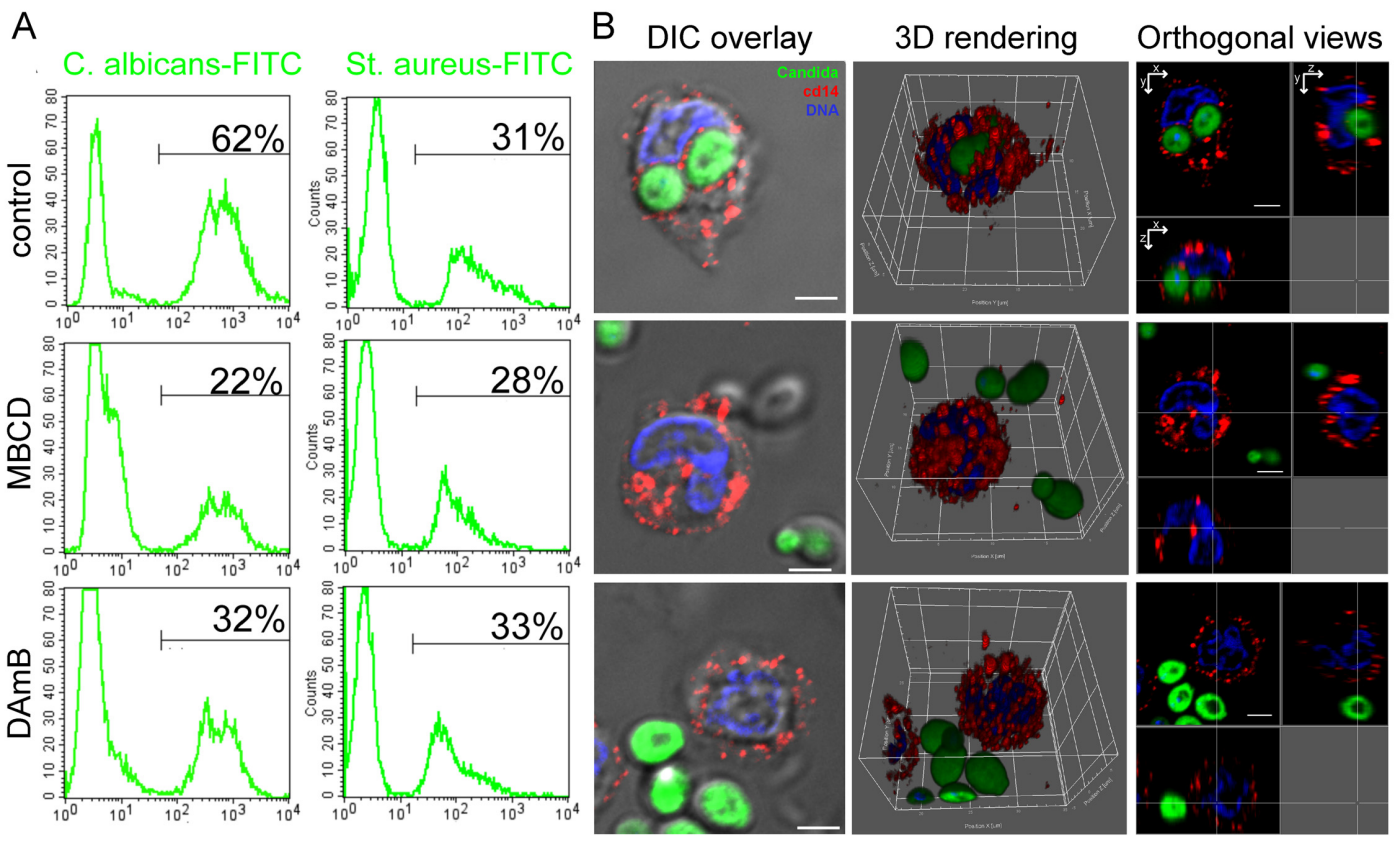
Disruption of lipid rafts inhibits *C*. *albicans* phagocytosis by human monocytes. **A.** Phagocytosis inhibition assessed by flow cytometry. Human monocytes were pretreated with MBCD, DAmB or left untreated (none), and then incubated with HK FITC-conjugated *C. albicans* or *Staphylococcus aureus* (*St. aureus*). The data highlight that drugs specifically impair fungal phagocytosis. Markers were set up to exclude background staining of cells incubated with the microorganisms at 0°C. Numbers indicate the percentage of monocytes with ingested microorganisms. Data shown are from one experiment representative of four. **B.** Phagocytosis analysis by confocal microscopy. Phagocytosis was performed as in A. Samples were acquired in 3D stacks by DIC and three-color fluorescent images. The triple staining allows to clearly establish if a FITC-conjugated *C*. *albicans* (green) was internalized by a monocyte labelled by anti-CD14 (red). DNA was counterstained with DAPI (blue). The first column (DIC overlay) shows the combined signals from the DIC and confocal fluorescent channels of a single xy slice. In the second column (3D rendering), the 3D reconstructions of the 22 slices highlighting the reciprocal spatial information of fungal and human cells are displayed. The third column (orthogonal views) shows the xy, xz and yz single planes at the indicated orthogonal xyz axes (white lines). Scale bar for all panels is 3 μ.

Data in the literature support the view that lipid rafts participate in the process of internalization of many different bacterial or parasitic pathogens by phagocytic cells, but there are no direct information on whether they also play a role in phagocytosis of the fungal pathogen *C*. *albicans* [11, 15, 16, 34]. To investigate this issue, we addressed lipid raft disruption by cholesterol-interfering treatments [35] in human peripheral blood CD14^+^ monocytes and evaluated whether this affected their ability to internalize *C*. *albicans* cells. In these experiments we treated peripheral blood CD14^+^ monocytes with two different raft-perturbing agents acting by distinct mechanisms: MBCD, a small cyclic oligosaccharide that selectively extracts cholesterol from the plasma membrane, and DAmb, a polyene antifungal agent that specifically binds and sequesters cholesterol [36]. Flow cytometry analysis (Fig 1A) showed that in the presence of MBCD, there was a significant reduction of the percentage of monocytes that ingested heat-killed, FITC-labeled fungal cells (22% versus 62% of the untreated control). We also found that sequestration of cholesterol by DAmb, without physical depletion, was sufficient to inhibit *C*. *albicans* phagocytosis by monocytes, since the effect of DAmb closely resembled what observed upon MBCD treatment in terms of percent reduction of the number of monocytes with ingested *C*. *albicans* cells (32% versus 62% of the untreated control, Fig 1A). Drugs treatment also inhibited phagocytosis of live *C*. *albicans* cells excluding that heat killing of the fungus could have modified the mechanism of internalization (data not shown). In contrast, lipid-raft disrupting agents did not affect the uptake of *Staphylococcus aureus* (Fig 1A), excluding the possibility of non-specific effects of the drugs on the phagocytosis process.

To confirm results from FACS experiments we performed immunofluorescence and confocal imaging. After the phagocytosis assay with HK FITC-conjugated *C*. *albicans* monocytes were fixed and stained for CD14, to label cellular boundaries, and with DAPI to visualize the nucleus. Confocal three-color imaging in 3D clearly differentiated between *C*. *albicans* cells internalized or in close proximity to the monocytes and confirmed phagocytosis inhibition by drugs treatments. Representative images of the three conditions are shown in Fig 1B where the acquired stacks were reconstructed to generate 3D images and orthogonal views of control untreated monocytes (upper row), MBCD and DAmb treated cells (central and lower row). Collectively, these experiments clearly indicate that lipid raft integrity maintained by cell membrane cholesterol is crucially required for *C*. *albicans* entry into human monocytes and suggest a novel route for host-fungi interaction. Moreover our data highlight an unexpected inhibitory effect of DAmb on fungal phagocytosis that, mimicking the effect of MBCD, can be related to its cholesterol-binding capacity.

### 2. *C*. *albicans* triggers the organization of a Dectin-1/lipid raft platform that drives its internalization by human monocytes

Several PRRs are known to mediate *C*. *albicans* recognition by myeloid cells [24, 25, 37–39]. We therefore set out to assess in human monocytes the expression of the major fungus-specific PRRs and their role in *C*. *albicans* internalization.

**Fig 2:**
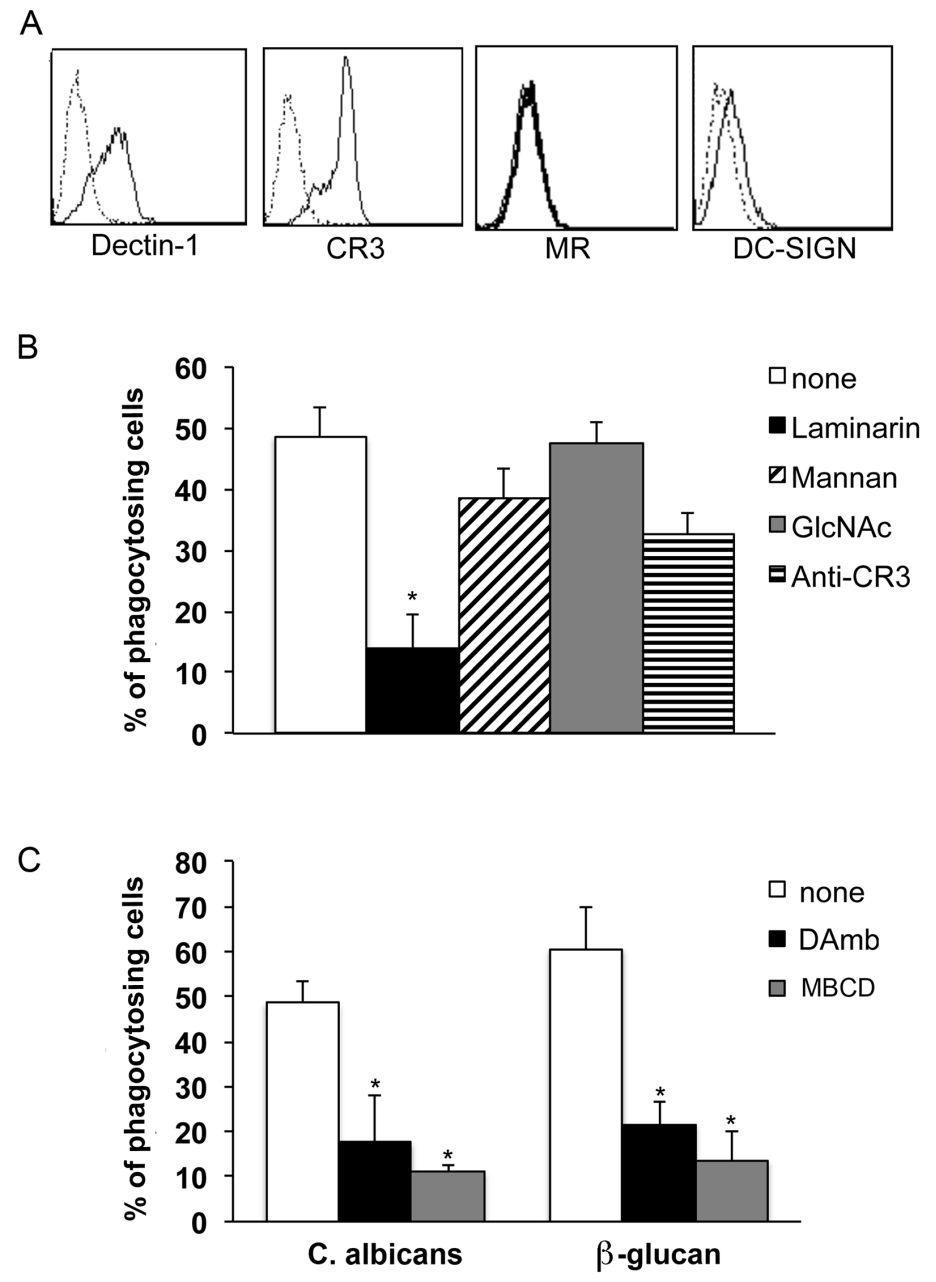
Dectin-1 is the predominant PRR involved in phagocytosis of *C*. *albicans* by human monocytes. **A**. Flow cytometry analysis of monocyte surface expression of *C*. *albicans*-specific PRRs. Circulating human monocytes express Dectin-1 and CR3 receptors but not MR and DC-SIGN. The appropriate isotype control for each mAb is reported as histogram with dotted lines. One experiment representative of five is shown. **B**. Inhibition of *C*. *albicans* phagocytosis by blocking *C*. *albicans-*specific PRRs. Monocytes were pre-treated with laminarin, mannan, acetylglucosamine (GlcNAc) to block Dectin-1, Dectin-2 or CR3, respectively or with a specific antibody anti-CR3, before incubation with FITC-conjugated *C*. *albicans*. Only laminarin impairs fungus phagocytosis, supporting the relevant role of Dectin-1 on *C*. *albicans* uptake. The asterisk indicates values significantly different from untreated monocytes (*p*<0.05). Results are the mean ± standard error (SE) of three independent experiments performed. **C.** Inhibition of the uptake of the specific Dectin-1 ligand β-glucan following monocyte lipid rafts disruption. Monocytes were pre-treated or not with DAmb or MBCD and then incubated with HK FITC-conjugated *C*. *albicans* or FITC-conjugated β-glucan. The extent of phagocytosis was assessed by flow cytometry. The uptake reduction was similar for both β-glucan and *C*. *albicans* suggesting the involvement of lipid rafts in Dectin-1 mediated phagocytosis. The asterisk indicates values significantly different (*p<*0.05) from untreated monocytes. Results are expressed as mean ± SE of three independent experiments.

As shown in Fig 2A, Dectin-1 and CR3 were homogeneously expressed, while the Mannose Receptor and DC-SIGN were undetectable. The uptake of *C*. *albicans* was only slightly affected by the addition of N-acetyl-D-glucosamine, a carbohydrate that specifically blocks the *C*.*albicans*-recognizing, lectin-like domain of CR3 [40], or by mannan, that blocks Dectin-2, another major fungus-recognizing receptor expressed by monocytes [41], as well as by the blocking of CR3 with a specific monoclonal antibody (Fig 2B). In contrast, blocking of Dectin-1 with the soluble, low molecular weight β-glucan laminarin strongly decreased monocyte ability to ingest fungal cells (Fig 2B).

These results clearly indicate that Dectin-1 is the receptor prevalently involved in the non-opsonic phagocytosis of *C*. *albicans* by human monocytes.

Recently, Xu et al. demonstrated that Dectin-1 translocates to lipid rafts upon stimulation of murine dendritic cells with its ligand β-glucan, a pan-fungal cell wall polysaccharide [42].

To investigate whether the destabilization of lipid rafts could specifically affect Dectin-1-mediated phagocytosis, we measured the effect of MBCD and DAmb treatment on the monocyte ability to internalize purified β-glucan particles. Data in Fig 2C show that the uptake of β-glucan particles decreased significantly by disruption of lipid rafts. The observed percent reduction of monocytes internalizing β-glucan particles was 79% and 65% in the presence of MBCD or DAmb, respectively. Interestingly, these percentages were comparable to those observed using whole *C*. *albicans* cells: 77% and 65% in the presence of MBCD or DAmb, respectively.

**Fig 3:**
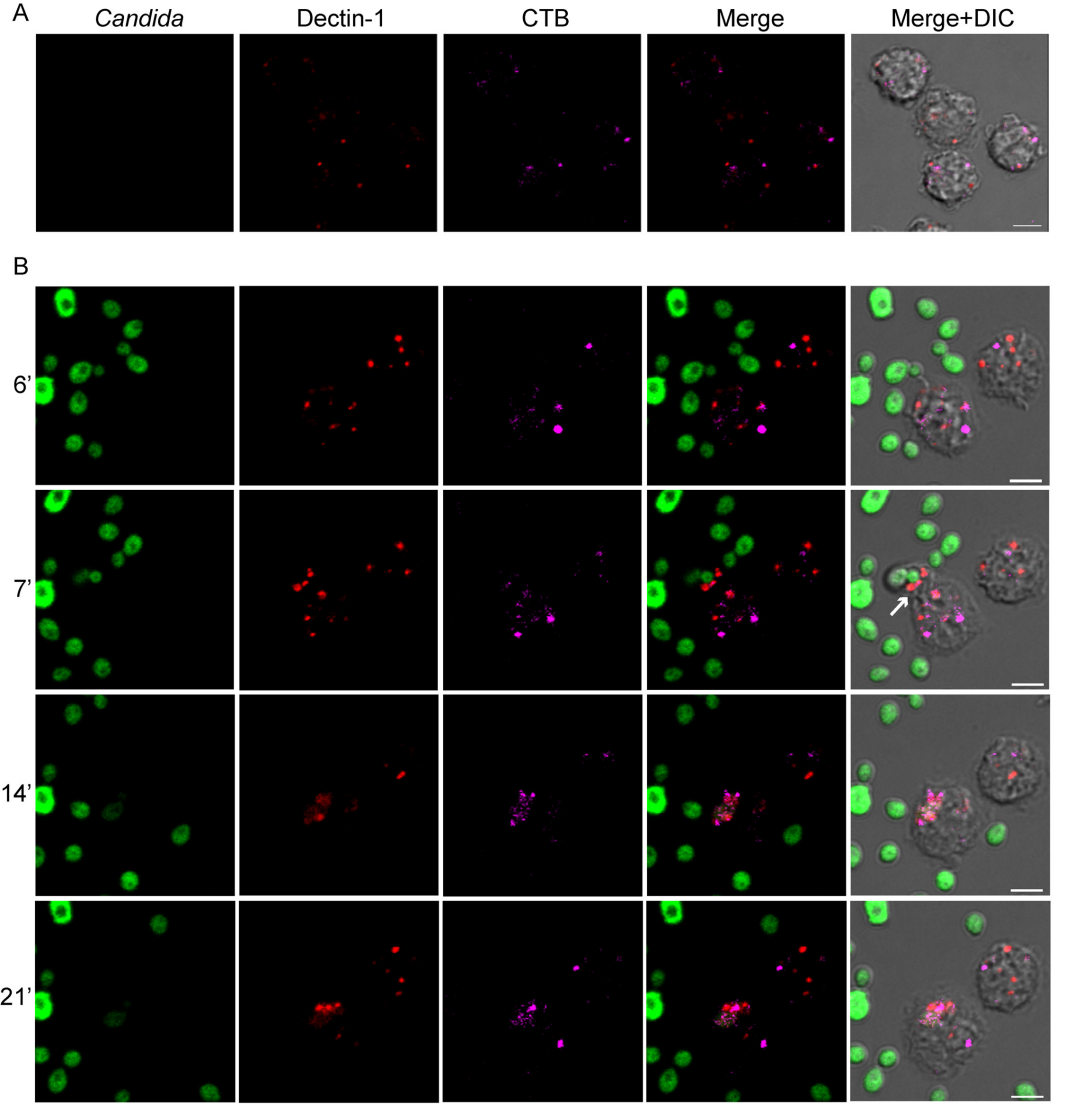
Involvement of lipid rafts in Dectin-1-mediated phagocytosis of *C*. *albicans*. Confocal time lapse analysis of Dectin-1 (red) and lipid rafts (CTB, magenta) dynamics in control, unstimulated human monocytes (**A**) and in FITC-*C*. *albicans* (green) stimulated human monocytes (**B**). Merge panels show the combined confocal fluorescent signals. The DIC images showing cell morphology are merged with the fluorescent channels in the last column. **A**. In absence of external stimuli, Dectin-1 receptor is scattered on the plasma membrane and no signal could be associated with the lipid rafts compartments. Scale bar is 5 μm. **B**. The timescale on the left indicates recording time. Soon after *C*. *albicans* stimulus, CTB signal starts to aggregate, indicating lipid rafts assembly in larger structures. With phagocytosis progression Dectin-1 association with lipid raft domains became stronger and clustering in correspondence of the point of monocyte-fungal cell contact increased and persisted during uptake and internalization. See also S1 Movie. Scale bars are 5 μm.

To provide a direct evidence of the involvement of lipid rafts in Dectin-1-mediated phagocytosis of *C*. *albicans*, we used time lapse and confocal three-color imaging to follow at single cell level fungus uptake and internalization by monocytes (Fig 3). To this aim, we stained live monocytes with a primary mAb anti-Dectin-1 plus an Cy3-conjugated goat-anti-mouse IgG secondary mAb and with AlexaFluor647-CTB that binds to the lipid raft marker GM-1. Monocytes were mixed for 30 minutes on ice with HK FITC-labelled *C*. *albicans* cells or left unstimulated and then plated on 8-wells μ-slide to be imaged at the confocal microscope for two hours to follow phagocytosis.

**Fig 4:**
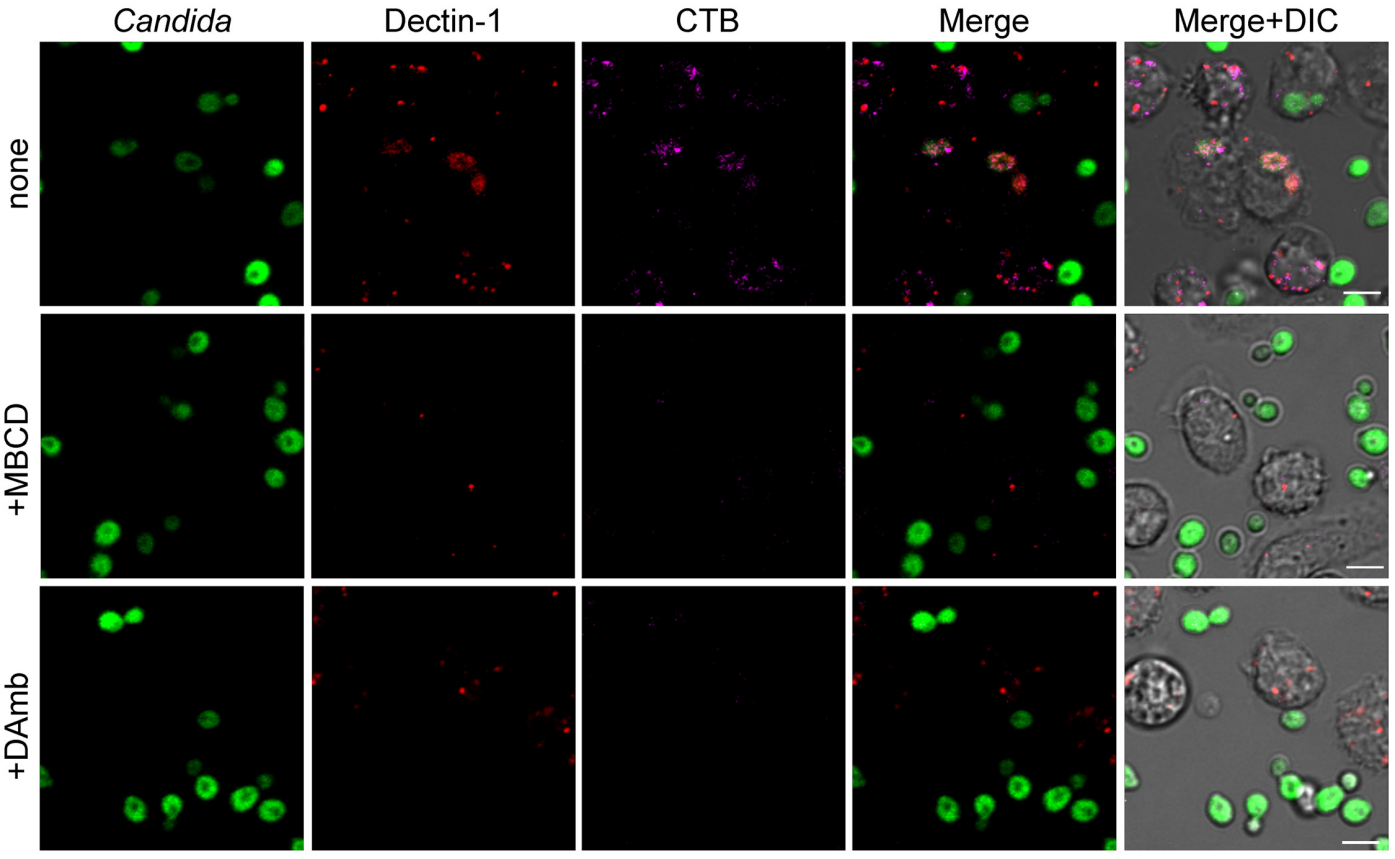
Effects of lipid raft perturbation on Dectin-1 dynamics during phagocytosis. Confocal time lapse analysis of Dectin-1 (red) and lipid rafts (CTB, magenta) dynamics in stimulated human monocytes after MBCD or DAmb treatment. Merge panels show the combined confocal fluorescent signals. The DIC images showing cell morphology are merged with the fluorescent channels in the last column. Scale bars are 5 μm. First lane are control cell not treated with drugs. MBCD or DAmb were added to the monocytes before fungi stimulation. Drugs treatment caused a complete disaggregation of lipid rafts (CTB channel). Dectin-1 polarization at the sites of *C*. *albicans* contact was strongly impaired. The resulting effect of these events was a severely decreased phagocytosis. See also Supplementary S2 and S3 Movies.

In unstimulated monocytes, shown in Fig 3A, lipid rafts (magenta) appeared as small, sparse dots and Dectin-1 receptors (red) were distributed in the plasma membrane, without any detectable association to lipid rafts, as shown in the merge panel. Time points of phagocytosis progression are displayed in Fig 3B (see also S1 Movie for the complete 2 hours experiment). Shortly after contact with fungal cells (green), CTB signal (magenta) converged into larger dots indicating monocyte lipid rafts coalescence and increased association with Dectin-1 (red) supported the receptor translocation into these broader membrane platforms (Fig 3B). With the progression of phagocytosis, lipid rafts and associated Dectin-1 were found to aggregate at the engulfing site during fungus uptake (Fig 3B, white arrow indicate a structure resembling the phagocytic cup at 7 minutes recording time; S1 Movie). During internalization the association became even stronger and persisted for all the duration of the experiment (Fig 3B, at 14 and 21 minutes and S1 Movie). Moreover, with the same dynamics, the observed monocyte engulfed another cell one hour after (middle of S1 Movie),

With similar experiments, we then treated non-fixed, live monocytes with MBCD and DAmb to observe how lipid raft perturbation would affect Dectin-1 dynamics upon interaction with *C*. *albicans* (Fig 4). This analysis showed that both MBCD and DAmb caused a complete disaggregation of lipid rafts, as demonstrated by the absence of coalescent magenta signal in drug-treated monocytes (Fig 4, CTB channel and S2 and S3 Movies). Dectin-1-enriched sites did not develop after *C*. *albicans* contact and the receptor was more discretely distributed on the plasma membrane similarly to unstimulated monocytes (Fig 3A), although its surface expression analysed by flow cytometry was not affected by drug treatments (data not shown). Phagocytosis appeared dramatically decreased (Fig 4, last column and S2 and S3 Movies), in agreement with our previous flow cytometry and microscopy data.

Results shown in Figs 3 and 4 prove the importance of analyzing live monocytes by time-lapse experiments to appreciate alterations of such a dynamic process as the organization of raft microdomains in the cell membrane. Taken together, these results demonstrate that the establishment of a functional lipid raft platform is essential for the process of *C*. *albicans* non-opsonic phagocytosis by human monocytes and for Dectin-1 dynamics of recruitment at the contact site enabling the formation of a committed phagocytic cup. [43]. The nature of this platform is apparently distinct from that involved in LacCer-mediated phagocytosis by neutrophils, since this latter is largely insensitive to cholesterol depletion or interference [44–46].

In consideration of the neat predominance of Dectin-1 in driving *C*. *albicans* uptake by monocytes, these results also strongly suggested that the inhibition of fungus phagocytosis observed upon raft interference was directly related to the ensuing dysfunctions of Dectin-1 membrane trafficking.

### 3. Raft disruption does not affect *Candida*-driven cytokine response by monocytes but drastically dampens their ability to induce a *C*. *albicans* specific T cell response

**Fig 5:**
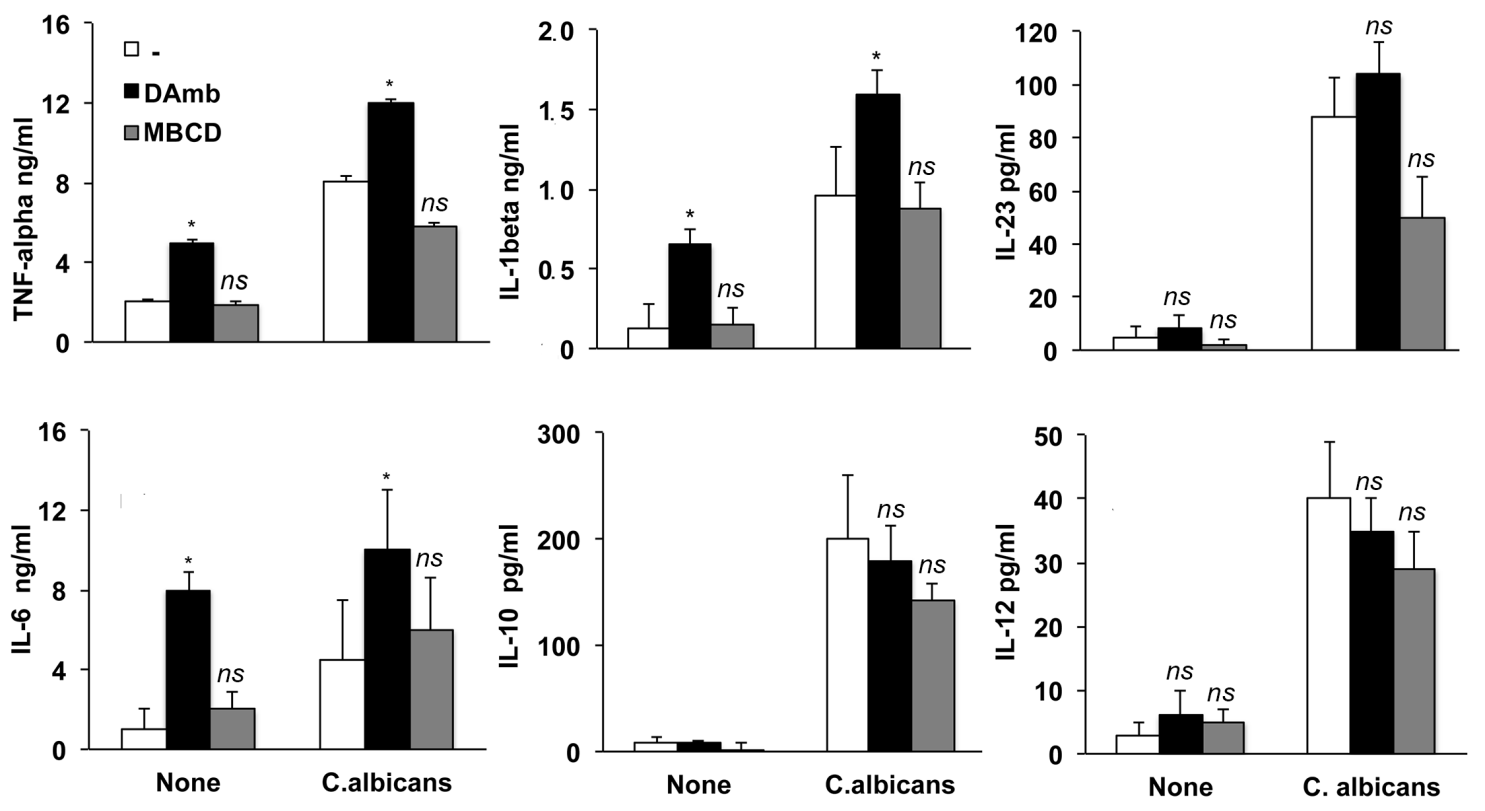
Disruption of lipid rafts does not modify cytokine production by monocytes in response to *C*. *albicans*. Monocytes, pretreated or not with DAmb or MBCD, were incubated with or without *C*. *albicans*. Supernatants were collected and cytokine levels were evaluated by ELISA. Histograms indicate mean values ± SE of three independent experiments. The asterisk indicates values (*p<*0.05) significantly different from untreated monocytes. *ns*: not significant.

We then investigated whether lipid raft disruption and consequent inhibition of *C*. *albicans* phagocytosis would affect other functional activities of monocytes. As shown in Fig 5, treatment with MBCD was unable to significantly alter either the basal or the *C*. *albicans*-induced production of inflammatory, and immunostimulatory cytokines. In contrast, treatment with DAmb caused *per se* the secretion of the inflammatory cytokines IL-1β, TNF-α and IL-6 by monocytes and synergized with *C*. *albicans* stimulation for IL-1β, TNF-α and IL-6 secretion (Fig 5). This data are in line with previous findings demonstrating the ability of DAmb to induce the production of pro-inflammatory cytokines and chemokines by THP-1 cells and human monocytes [47–49]. However, DAmb pretreatment had no effect on the basal or the *Candida*-stimulated production of the T cell-polarizing cytokines IL-12 and IL-23 and of IL-10 (Fig 5).

It was evident from these data that *C*. *albicans* could trigger the release of diverse cytokines irrespective of lipid raft integrity and efficient internalization by monocytes.

Uncoupling between phagocytosis and cytokine expression is in agreement with previous results by other authors showing that internalization of *C*.*albicans* cells or fungus-mimicking particles by phagocytes is dispensable for the induction of several cytokines [50–54]. Moreover, with respect to the Dectin-1 receptor, it is known that signaling pathways leading to phagocytosis or cytokine expression are distinct [55]. On the other hand, the finding that lipid raft integrity is irrelevant for cytokine induction by *C*. *albicans* is in apparent contrast with the notion that several PRRs require to be organized in lipid rafts to initiate cytokine signaling upon agonist binding [12, 56, 57]. It should be considered, however, that the fungus-monocyte interaction we analyzed is extremely complex as compared to the interaction of a single receptor with its ligand in soluble form. It is conceivable that the simultaneous engagement of different monocyte receptors by several *C*. *albicans* PAMPs, gathered together and highly repeated on fungal cell surface, could bypass lipid rafts and facilitate *per se* the co-localization and integration of different signaling components triggered also by fungus binding in the absence of phagocytosis.

**Fig 6:**
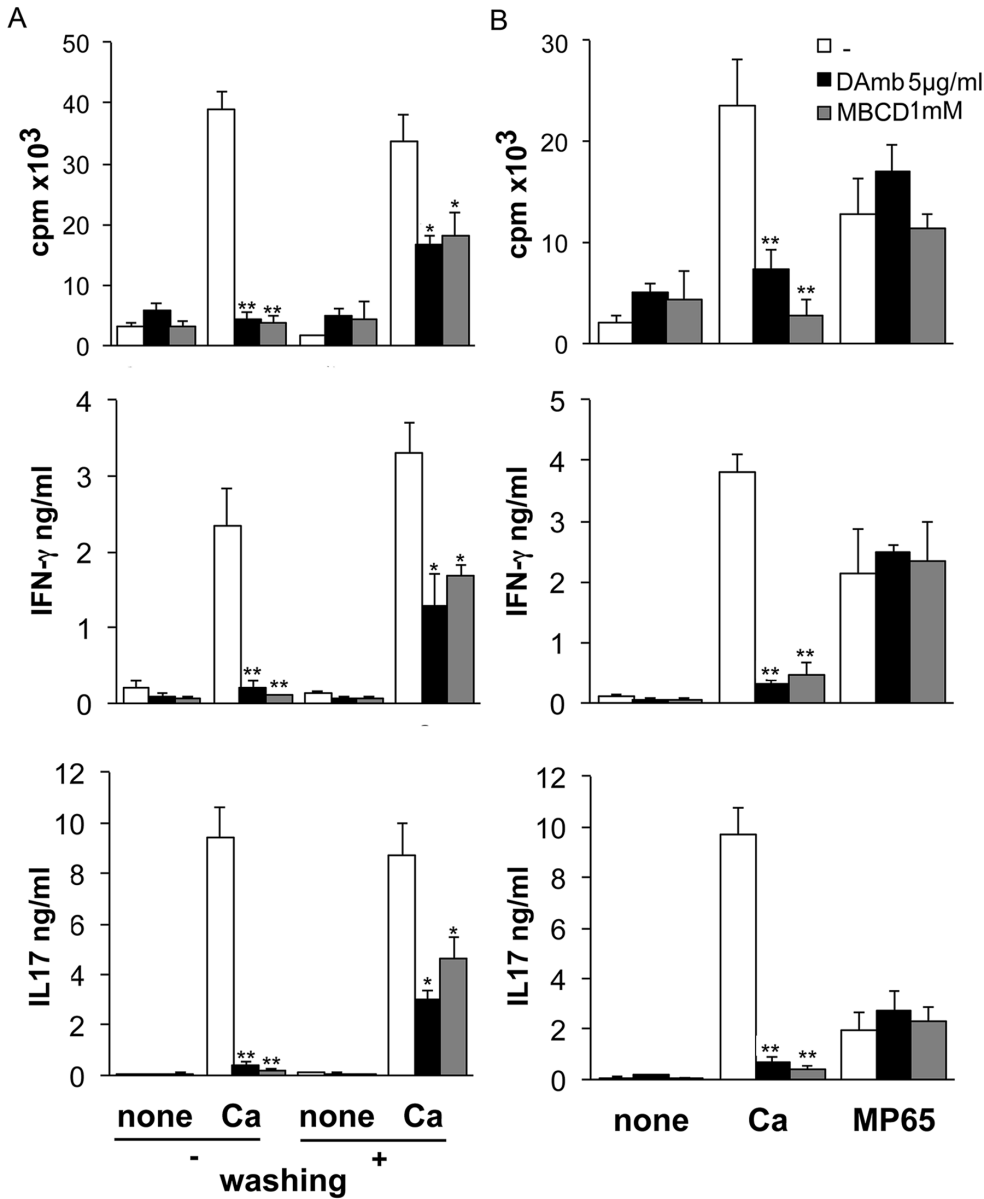
Disruption of lipid rafts blunts T cell response *to C*. *albicans*. Monocytes, treated or not with DAmb or MBCD before incubation with HK *C*. *albicans* (Ca) or with MP65, were co-cultured with autologous memory CD4^+^ T lymphocytes for 6 days. At day 6 cell proliferation was measured by thymidine incorporation, expressed as count per minutes (cpm), and culture supernatants were harvested for cytokine production analysis by ELISA. **A**. Part of the monocytes treated with drugs and pulsed with *C*. *albicans* were extensively washed or not before incubation with T cells (+/- washing). Drugs removal only partially restored T cell response supporting a major effect on monocytes. **B**. T cell response to MP65, which does not require phagocytosis for its uptake and processing, was left unmodified by the presence of drugs excluding a non specific effect on antigen processing and/or presentation by monocytes. Asterisks indicate values significantly different from non-treated monocytes: (*p<0.05; **p<0.001). One experiment out of two is shown as means ± SD of triplicate wells.

Phagocytosis of a pathogen by APC is required to permit antigen processing and presentation to specific T cells. We thus investigated the impact of raft disruption and the consequent decreased in fungus uptake by monocytes on the magnitude and/or quality of specific memory CD4+ T cell responses in human donors. Monocytes isolated from donors highly responsive to *C*. *albicans* were pulsed with *C*. *albicans* in the presence of DAmb or MBCD for 2 hours, and then autologous purified memory CD4+ T cells were added. As shown in Fig 6A memory CD4+ T cells stimulated by untreated monocytes exhibited, as expected, a robust proliferative response and cytokine production in response to the fungus. On the contrary, the same population of T cells barely proliferated and produced IFN-γ and IL-17 in response to drugs treated *C*. *albicans*-pulsed monocytes. The impairment of T cell response to *C*. *albicans* following lipid raft disruption might be ascribed to several mechanisms other than the decreased fungus uptake. First, to evaluate whether the decreased T cell response might result from a direct drugs effect on T cells, treated monocytes were extensively washed after antigen pulse and then co-cultured with T cells (Fig 6A). Drugs removal only partially restored T cell response, reinforcing the hypothesis that drug's effects on monocytes are the main cause for the reduced T cell activation. Moreover the observed partial rescue of T cell proliferation could also be due to the phagocytosis of residual *C*. *albicans* cells, which cannot be totally removed from the culture by washings. According with these results, the memory CD4+ T cell response to MP65, a purified, antigenic *Candida albicans* protein (Fig 6B), which does not require phagocytosis for its uptake and processing, was left unmodified by the presence of drugs. These results also excluded that drugs could generically blunt antigen processing and/or presentation by monocytes.

Altogether our data suggest that inhibition of *C*.*albicans*-specific T cell responses by the lipid raft-disrupting agents might directly ensue, at least for the most part, from the drug ability to inhibit the phagocytosis of the fungus by monocytes, reducing the antigen load available for antigen presentation. This interpretation is in line with previous reports by other authors that postulated, in different microbial models, a close association between extent of pathogen internalization by various antigen-presenting cells and their efficacy for evoking pathogen-specific T cell responses [58–61]. Moreover the above findings suggest that integrity of lipid raft platform on antigen presenting cells is crucial for the induction of antigen specific T cell responses to fungi.

## Conclusions

In this work we provide a first demonstration that lipid rafts critically contribute to the interaction of *C*. *albicans* with Dectin-1 and we suggest that they might provide a proper entry platform for other β-glucan-expressing fungi. Our results provide evidence that disruption of lipid rafts translates into an altered Dectin-1 dynamics in the cell membrane of antigen presenting cells and dramatically affects their efficiency to capture the fungal cells and to trigger an efficient, Th1- or Th17-type antifungal T cell responses.

A novel and unexpected finding originated by our study is also the evidence that DAmb can significantly impair immune recognition of *C*. *albicans*, and possibly other pathogenic fungi. This issue is of relevance, since DAmb remains even now a first line treatment for severe and life threatening systemic fungal infections, including candidosis [62, 63]. It is long known that this drug has strong proinflammatory and immunomodulatory effects, that are believed to contribute both to its protective and toxic effects [63], but the ability to inhibit phagocytosis and antigen specific T cell response has never been recognized before. It is largely unpredictable whether and to what extent DAmb could exert the effects we observed in our *ex-vivo* model in the *in vivo* situation. However, our findings open new questions about how DAmb acts during fungal infections besides its potent fungicidal activity.

## Supporting Information

S1 Movie Lipid rafts participate to Dectin-1-mediated phagocytosis of *C*. *albicans*. (MP4)

S2 Movie Treatment of monocytes with MBCD causes a complete lipid raft disaggregation and inhibition of fungal phagocytosis. (MP4)

S3 Movie Treatment of monocytes with DAmb causes a complete lipid raft disaggregation and inhibition of fungal phagocytosis. (MP4)
